# Supplementary material for: Novel insights from comprehensive analysis: The role of cuproptosis and peripheral immune infiltration in Alzheimer’s disease
Source: PLoS One. 2025 Jun 25;20(6):e0325799. doi: 10.1371/journal.pone.0325799 (PMC12194219; doi:10.1371/journal.pone.0325799)

Figure S1. Gene set enrichment analysis for the five genes in AD patients. KEGG pathway analysis revealed significant biological processes in the high and low expressions of FDX1 (A), MAP2K1 (B), PDK1 (C), GLS (D), and SOD1 (E).


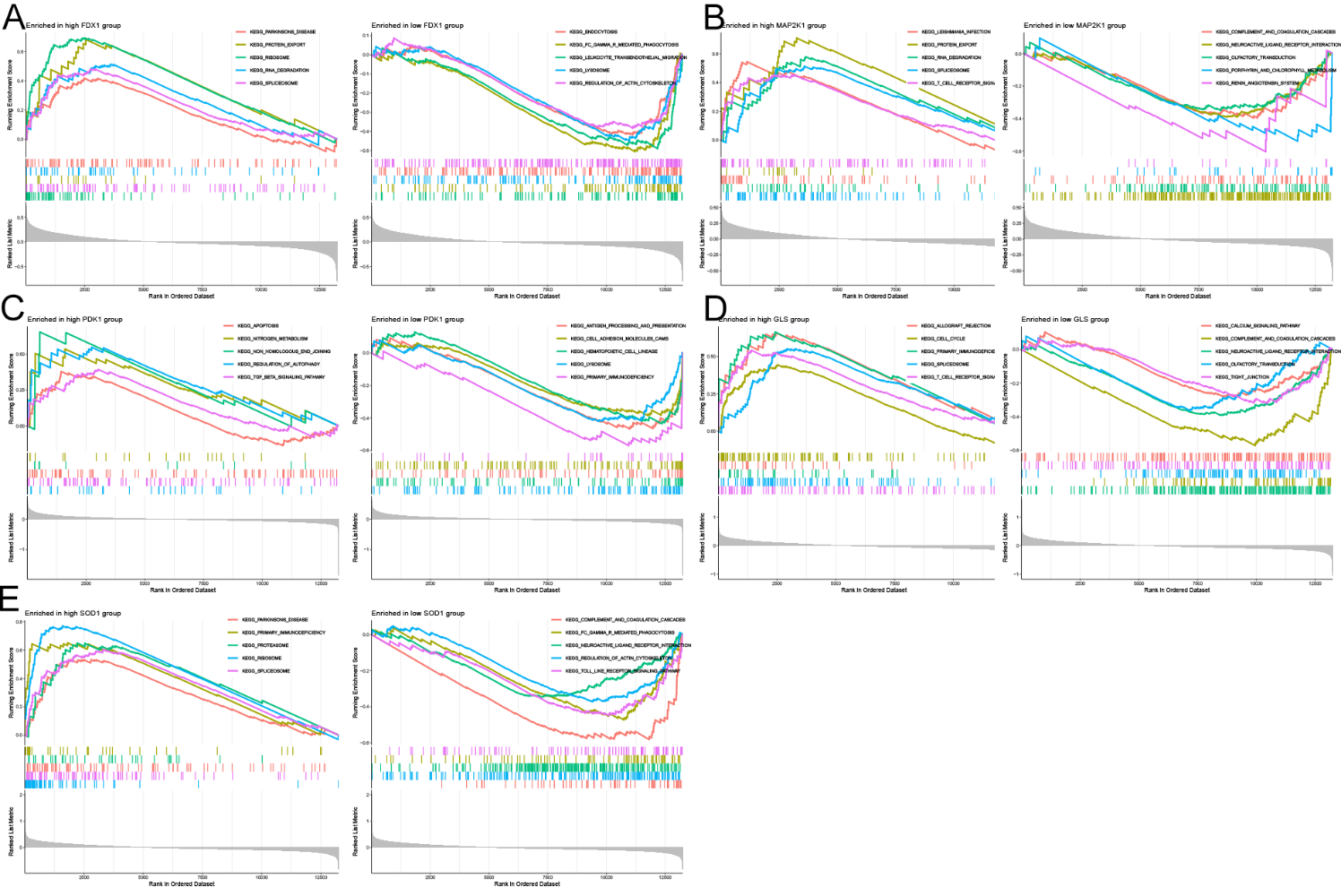

Supplement: S1 Fig — KEGG pathway analysis revealed significant biological processes in the high and low expressions of FDX1 (A), MAP2K1 (B), PDK1 (C), GLS (D), and SOD1 (E). (DOCX) [file pone.0325799.s001.docx]
