# Supplementary material for: Novel insights from comprehensive analysis: The role of cuproptosis and peripheral immune infiltration in Alzheimer’s disease
Source: PLoS One. 2025 Jun 25;20(6):e0325799. doi: 10.1371/journal.pone.0325799 (PMC12194219; doi:10.1371/journal.pone.0325799)

Figure S2. Single-cell analysis and cell-cell communication. (A) Quality control of the GSE181279 dataset. (B) Scatter plot of the correlations of sequencing depth. (C) Elbow plot of the principal components in PCA. (D) PCA plot showing the downscale results. (E) The feature genes of each principal component. (F) Heatmap of the PCA feature genes. (G) A t-SNE plot visualizing cell annotation. (H) A bubble plot demonstrating typical cell surface markers defining five immune cell types, including CD4+ T cells, NK cells, B cells, CD8+ T cells, and monocytes. (I) Volcano plots of DEGs in five immune cell types between AD patients and healthy controls. (J) Scatter plots demonstrating the distribution of hub-genes in cells of the AD and Control groups. (K) A bubble plot demonstrating the percentage expression of hub-genes in each immune cell in the AD and Control groups. (L) A heatmap illustrating the MIF signaling network. (M) A violin plot demonstrating the gene expression levels of the MIF signaling pathway. (N) The contribution of the MIF signaling pathway.


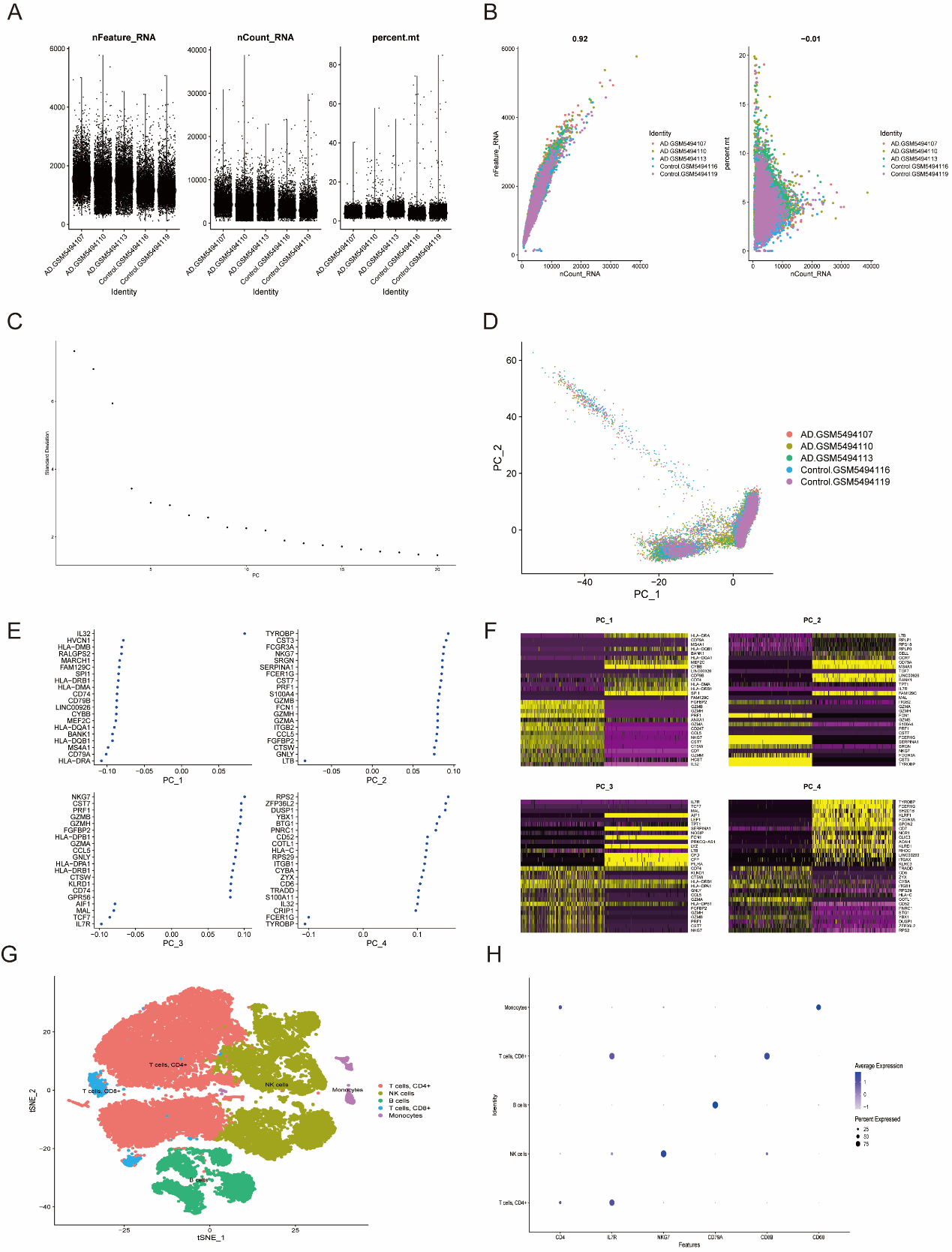


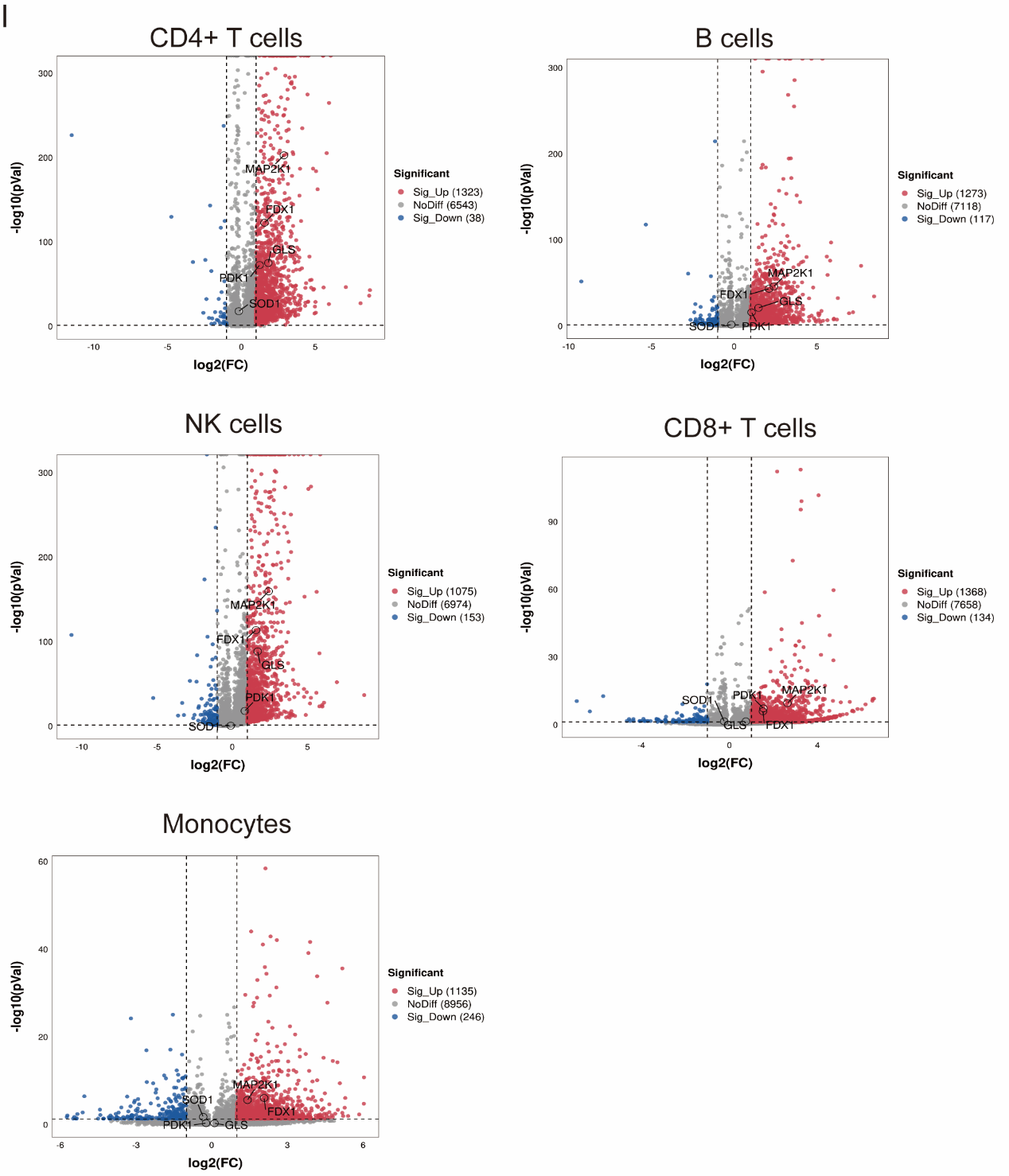


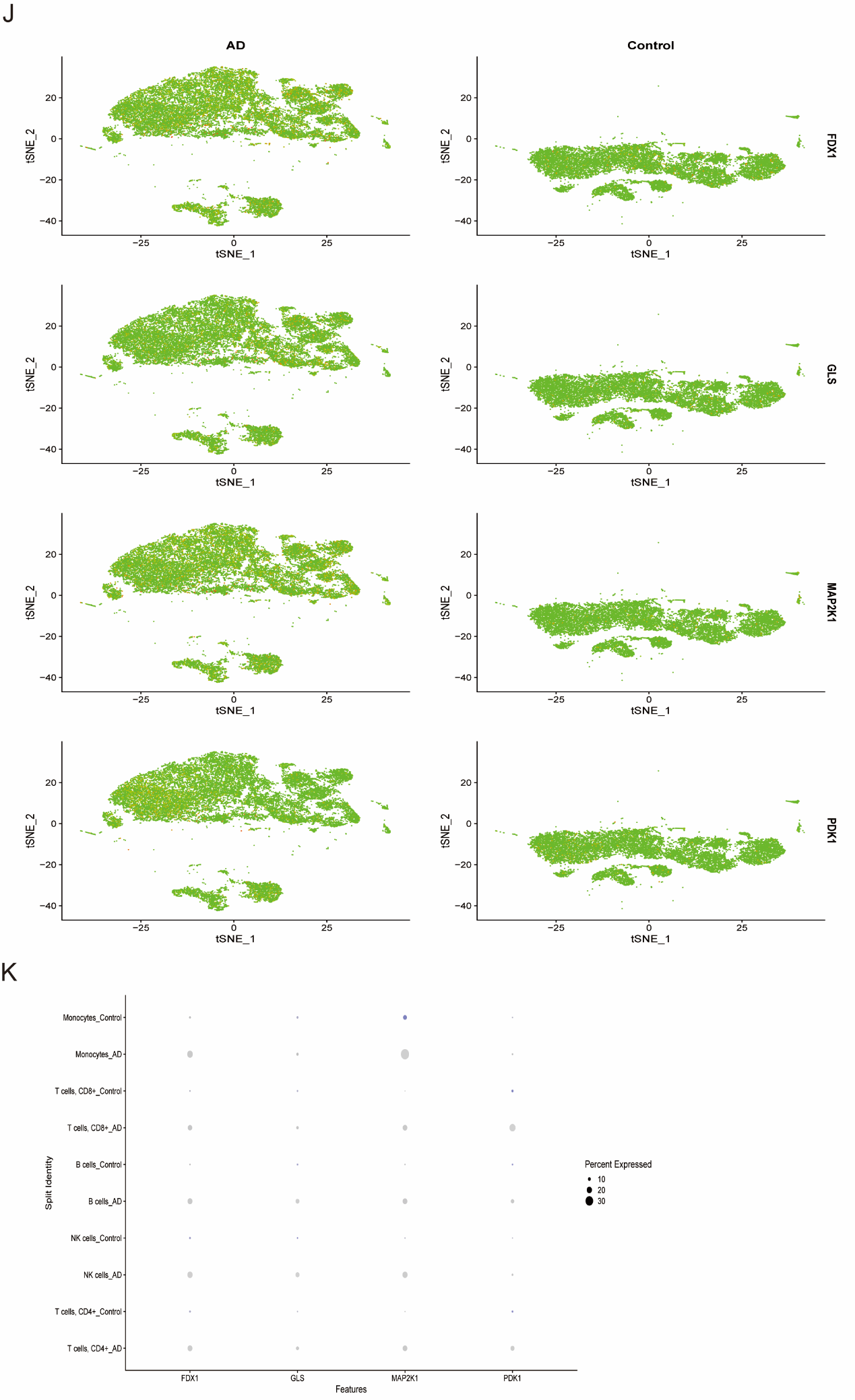


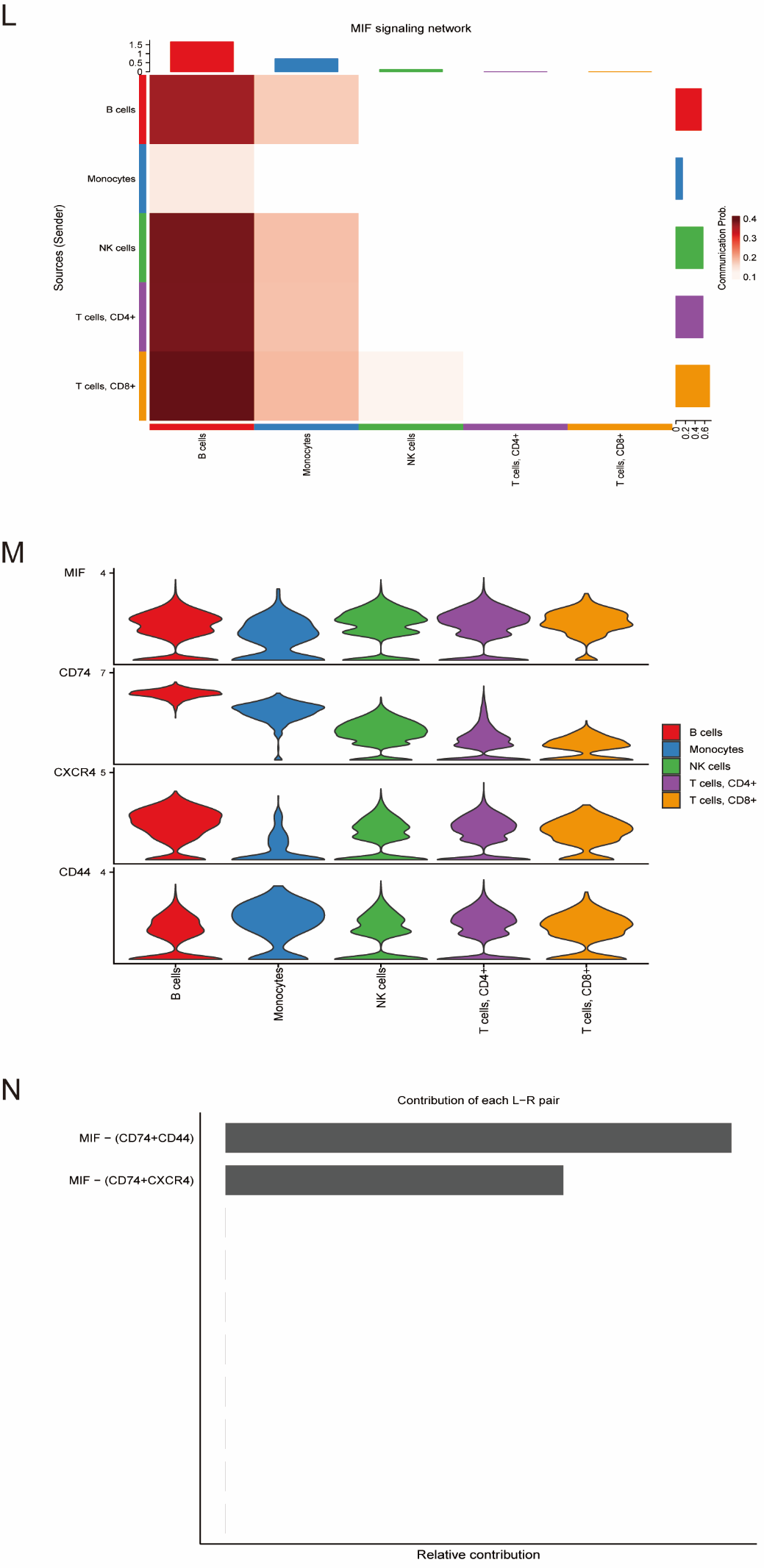

Supplement: S2 Fig — (A) Quality control of the GSE181279 dataset. (B) Scatter plot of the correlations of sequencing depth. (C) Elbow plot of the principal components in PCA. (D) PCA plot showing the downscale results. (E) The feature genes of each principal component. (F) Heatmap of the PCA feature genes. (G) A t-SNE plot visualizing cell annotation. (H) A bubble plot demonstrating typical cell surface markers defining five immune cell types, including CD4+ T cells, NK cells, B cells, CD8+ T cells, and monocytes. (I) Volcano plots of DEGs in five immune cell types between AD patients and healthy controls. (J) Scatter plots demonstrating the distribution of hub-genes in cells of the AD and Control groups. (K) A bubble plot demonstrating the percentage expression of hub-genes in each immune cell in the AD and Control groups. (L) A heatmap illustrating the MIF signaling network. (M) A violin plot demonstrating the gene expression levels of the MIF signaling pathway. (N) The contribution of the MIF signaling pathway. (DOCX) [file pone.0325799.s002.docx]
