# Supplementary figures and images for: Novel insights from comprehensive analysis: The role of cuproptosis and peripheral immune infiltration in Alzheimer’s disease
Source: PLoS One. 2025 Jun 25;20(6):e0325799. doi: 10.1371/journal.pone.0325799 (PMC12194219; doi:10.1371/journal.pone.0325799)

Figure S3. Amplification and lysis curves for each tested gene. (A) *SOD1*. (B) *FDX1*. (C) *PDK1*. (D) *GLS*. (E) *MAP2K1*. (F) *GAPDH*.


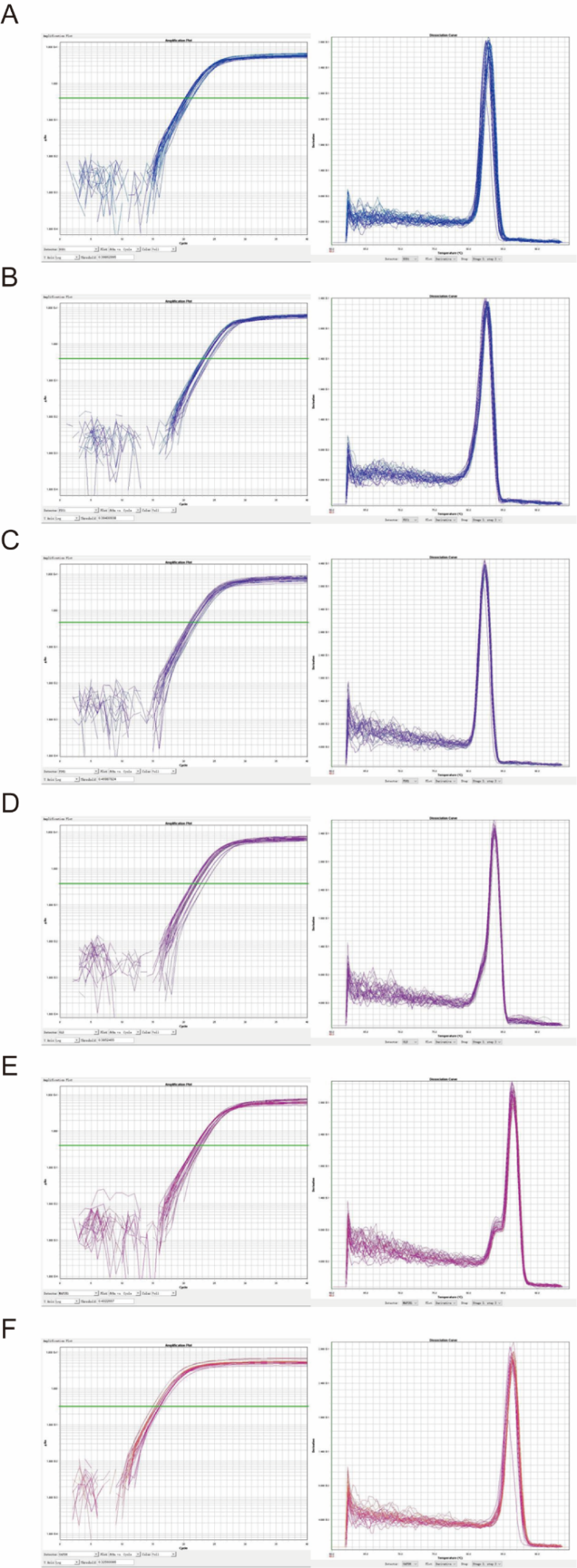

Supplement: S3 Fig — (A) SOD1. (B) FDX1. (C) PDK1. (D) GLS. (E) MAP2K1. (F) GAPDH. (DOCX) [file pone.0325799.s003.docx]
