# Supplementary material for: Novel insights from comprehensive analysis: The role of cuproptosis and peripheral immune infiltration in Alzheimer’s disease
Source: PLoS One. 2025 Jun 25;20(6):e0325799. doi: 10.1371/journal.pone.0325799 (PMC12194219; doi:10.1371/journal.pone.0325799)
Supplement: S1 Table — (DOCX) [file pone.0325799.s004.docx]

Supplementary Table 1. The specific information of datasets in this study.

| **Dataset** | **Platform** | **Normal samples** | **AD samples** | **Type** |
| --- | --- | --- | --- | --- |
| GSE181279 | GPL24676 (Illumina NovaSeq 6000) | 2 | 3 | Single-cell RNA (whole blood) |
| GSE63060 | GPL6947 (Illumina HumanHT-12 V3.0 expression beadchip) | 104 | 145 | Messenger RNAs (whole blood) |
| GSE33000 | GPL4372 (Rosetta/Merck Human 44k 1.1 microarray) | 157 | 310 | Messenger RNAs (cerebral cortex) |
| GSE122063 | GPL16699 (Agilent-039494 SurePrint G3 Human GE v2 8x60K Microarray 039381) | 44 | 56 | Messenger RNAs(cerebral cortex) |

AD, Alzheimer's disease
