# Supplementary material for: Novel insights from comprehensive analysis: The role of cuproptosis and peripheral immune infiltration in Alzheimer’s disease
Source: PLoS One. 2025 Jun 25;20(6):e0325799. doi: 10.1371/journal.pone.0325799 (PMC12194219; doi:10.1371/journal.pone.0325799)
Supplement: Attachment — Submitted filename: Response to Reviewers.docx [file pone.0325799.s014.docx]

Dear Editor and Reviewer,

On behalf of my co-authors, we are very grateful to you for giving us the opportunity to revise our manuscript, thank you very much for your positive and constructive comments on the manuscript. All journal requests were taken seriously, including that we have removed the Funding section in our manuscript and we have stated the work funders took in the Author’s contributions section (Lines 690-695). And the Ethics approval and consent to participate section has been removed and the related content has been moved to Lines 277-280 in the Methods section.

After carefully reading your comments and suggestions, we have revised and responded according to them and displayed them below:

**Reviewer #1**

Q1. I have found some similar studies: https://www.frontiersin.org/journals/molecular-biosciences/articles/10.3389/fmolb.2024.1478611/full. which closely resembles the figure type presented in this manuscript. Additionally, other related studies include:
1. <https://www.frontiersin.org/journals/aging-neuroscience/articles/10.3389/fnagi.2022.932676/full>

2. <https://www.frontiersin.org/journals/aging-neuroscience/articles/10.3389/fnagi.2023.1204530/full>

Moreover, I noticed that the dataset and methodology used in this manuscript appear to be the same as in previous studies. The authors should establish a strong hypothesis and present a novel approach to distinguish their work.
Response: Thank you for bringing these related studies to our attention and for the constructive feedback regarding the similarity. We would like to address these points as follows:

Similarity in Figure Formats: While our figure design may appear similar to those in the referenced studies, the chosen format was selected to clearly and effectively communicate our specific findings. We think that standard visual representations help facilitate comparisons across studies. Moreover, the content in these pictures is completely different from other studies, including the enrichment analysis, GSVA analysis in the two Clusters, machine-learning models, single-cell analysis, cellular communication, and Pet-CT results.

Dataset and Methodological Overlap: We acknowledge that our dataset and certain methodological elements share similarities with prior work in this area. However, our study differs in several important respects: **a.** Unlike previous studies, we have incorporated single-cell analysis to a stronger, more targeted hypothesis regarding cuproptosis involved in AD progression. **b.** The number of CAGs included in this study was greater than other studies, and we employed four machine-learning methods to develop a cuproptosis-related model, which was distinguished from other work. **c.** Our interpretation of the results diverged from that in the referenced studies. We provided a detailed discussion on how our findings contribute to a deeper understanding of MIF pathway and intracellular communication and the potential implications for future research. We have enhanced our methodological description to emphasize the novel aspects of our approach and how these innovations contribute to a more robust understanding of the underlying biological questions. Please refer to the revised content of Methods, Results, and Discussions in the manuscript.

Q2. The authors have utilized array datasets rather than sequencing. Since sequencing offers an advantage over microarrays in detecting novel transcripts, was sequencing data unavailable?

Response: Thank you very much for your significant question. We acknowledge that RNA-seq offers distinct advantages, particularly in its ability to detect novel transcripts. However, our primary objective was to analyze expression patterns of cuproptosis-associated genes across a large, clinically robust cohort. The selected array datasets provided extensive metadata, high reproducibility, and sufficient coverage of established transcripts, making them ideally suited to our study’s focus on differential expression rather than novel transcript discovery. The microarray datasets available to us were generated under highly standardized conditions, ensuring uniformity across samples. This consistency, coupled with a substantial sample size, allowed us to perform rigorous statistical analyses.

Moreover, we employed a high-throughput sequencing dataset, namely the GSE181279 scRNA-seq dataset, to validate our results in single-cell levels.

Q3. PCA was conducted on DECAGs, with only two clusters chosen for further analysis. Were these clusters selected based on a specific p-value or eigenvectors with the highest eigenvalues? Additionally, please specify the number of data points mapped to these two clusters.

Response: We sincerely appreciate the valuable feedback. We are sorry for our negligence. We have reintroduced the optimum cluster selection based on the consensus matrices and the cumulative distribution function (CDF) curve (Lines 173-174). The results are shown in Figures 5A- C, which indicate that the optimum number is k=2. PCA is performed to intuitively visualize the optimum clustering results (Lines 174-176). Figure 5D shows the points of the scatterplot denoting each sample of the two main components of the cluster (PC1 and PC2), which suggests the clustering analysis when k=2 is reasonable and stable. The number of data points is 90 (Cluster1) and 55 (Cluster2), respectively (Table S5, Lines 357-358). We hope these revisions adequately address your concerns.

Q4. For the machine learning models, 70% of the dataset was used for training, while 30% was reserved for testing. However, the authors have not mentioned the number of epochs or the cross-validation techniques applied. Furthermore, the dataset should ideally be divided into training, validation, and testing subsets. Please discuss.
Response: Thank you very much for pointing this out. We deeply apologize for this omission. We have added that we employed k-fold cross-validation (k=5) to ensure optimal selection in machine learning (Lines 200-201).

We have also added the process of dividing the AD patients in the GSE63060 dataset into a training cohort and a test cohort in Lines 185-186.

We validated the risk model using ROC curves on external datasets (GSE33000 and GSE122063). We have added the validation method in Lines 211-214 and revised the validation results in Lines 397-402.

We hope these revisions and clarifications address your concerns and illustrate the distinct contributions of our study. Thank you again for the opportunity to improve our manuscript.

**Reviewer #2**

Q1. The machine learning models were used to identify hub-genes, but the statistical significance of these findings is not adequately demonstrated. It remains unclear whether cross-validation was performed and how the performance of each model was evaluated. The manuscript mentions that XGB automatically selects the best features based on weights, but the criteria or thresholds used for feature selection are not specified. Although the manuscript provides general descriptions of the machine learning algorithms, a more detailed explanation of the specific configurations, parameters, and any custom modifications to the standard algorithms would be beneficial. For instance, how were the parameters for RF and SVM optimized? Furthermore, the manuscript mentions several R packages (e.g., "caret", "randomForest", "kernlab", "xgboost", "e1071", "rms"), which is useful for reproducibility, but the version numbers of these packages should be provided to avoid discrepancies in future replication attempts. Lastly, a detailed explanation of how points were assigned to each gene in the nomogram, as well as the calculation of total points, would be advantageous. The statistical methods used to assign these points should also be clearly outlined.

Response: We sincerely appreciate the constructive suggestions. We sincerely apologize for our negligence. In the revised manuscript, we have expanded the Methods section to provide additional details as follows:

**Cross-Validation and Performance Evaluation:**

We reintroduce the cross-validation methodology in Lines 200-201, stating that the optimal choice of the four machine learning algorithms is derived from 5-fold cross-validation with default parameters. Model performance was assessed using residual analysis, ROC curves (via the “pROC” package), and the “model_performance” function, ensuring that the statistical significance of our findings was adequately demonstrated (Lines 201-204).

**Feature Selection in XGB:**

The XGB model automatically selects features based on weight built-in algorithm (Lines 200-201), the Gradient Boosting Decision Tree (GBDT). Default criteria embedded within the xgboost package (version 1.7.8.1) were used to determine the optimal number of features. The feature importance was then further assessed using the “variable_importance” function to identify hub genes.

**Parameter Optimization for RF and SVM:**

By default, the maximum depth of the tree is 6 (max_depth = 6), the learning rate is 0.3 (eta = 0.3), the L2 regularization parameter is 1 (lambda = 1), the L1 regularization parameter is 0 (alpha = 0), and the number of iterations is 100 (nrounds = 100). All of the four models are calculated using built-in algorithms (Lines 196-199). Here, we list some important default parameters of these models as follows: (In the RF model, the default number of trees is 500 (ntree = 500) and the default minimum sample size of leaf nodes is 1 (nodesize = 1). In the SVM model, default regularization parameter is 1(C = 1). We used the “kernlab” (version 0.9.33) and “e1071” (version 1.7.16) packages to minimize classification errors using cross-validation. The models were run with default parameters, which were automatically tuned to find the best classification boundaries, and again 5-fold cross-validation was used to ensure model generalization capabilities. No additional customizations were made to this section.

Overall, all models were configured with default parameters based on 5-fold cross-validation, and no special customization was made. This ensures standardization of model training and facilitates repeated validation of the results.

**R packages versions:**

We added the version numbers in the “Construction of cuproptosis-related AD risk models” section. Please refer to Lines 198-199 and 208.

**Nomogram and statistical methods:**

We have added “Each of the five genes was given a different point value, and the points were summed to give the total points. If the total points did not exceed 140, the AD risk was less than 0.1, and if the total points exceeded 240, the AD risk was greater than 0.9.“ Please review Lines 391-394.

Thank you once again for your thoughtful review.

Q2. As for molecular docking, a more detailed explanation of the selection process would improve the transparency of the study.
Response: Thank you for your constructive suggestion regarding the selection of the predicted drugs. We apologize for the ambiguity of the article due to our negligence. Criteria for drug selection were based on interaction scores in gene-drug interaction networks constructed using the DGIdb database and available literature support rather than docking scores. DGIdb is a database that integrates drug-gene interaction data from multiple sources (e.g., rugBank, PharmGKB, TTD, and ChEMBL) and provides comprehensive scores for drugs based on experimental evidence, literature support, bioavailability, binding affinity, and more. We have now added the interaction scores of the candidate drugs for each hub-gene (Table S6, Line 427). Furthermore, we have included relevant references that support the selection of drug candidates based on interaction scores, as well as supporting literature. Please see Lines 230-232 and references 36-40 in the manuscript. We appreciate your attention to detail.

Q3. The manuscript reports that the binding energies of the selected compounds with their respective hub-gene related proteins are all below -7.0 kcal/mol, suggesting strong binding affinity. However, more context regarding this threshold would be helpful. Is this value supported by the literature, or is it specific to this study?

Response: Thank you for your valuable comment. We have now added more relevant references in support of this threshold that indicate strong binding affinity between ligand and receptor. Please see Line 440, references 45-49 in the manuscript. We appreciate your attention to detail.

Q4. While the docking results provide valuable structural insights, the biological relevance of these findings in the context of Alzheimer's disease (AD) is not fully addressed. How do the predicted binding affinities relate to AD treatment? Are there experimental studies or existing literature supporting the involvement of these proteins and interactions in AD? A deeper discussion of the biological significance of these results would enhance the manuscript's impact.

Response: Thank you for your valuable feedback, which suggests a deeper discussion of the molecular docking results and their relationship to AD treatment. We appreciate your insights and have revised the discussion section to be more comprehensive.

In our revised manuscript, we found literature supporting that the predicted binding affinity of a molecule to a specific biological target is critical for the development of AD therapeutics. Also, we concluded the reported possible relevance of the four compounds to AD treatment. For instance, aberrant activation of the MEK/ERK pathway is closely associated with the progression of AD. AZD8330, a newly discovered MEK1/2 inhibitor, may improve AD progression by blocking the MEK/ERK pathway, although direct evidence is lacking.

Please refer to the Lines 621-657 in the revised manuscript. We believe that these revisions significantly enhance the discussion and provide a more thorough context for our findings. Thank you once again for your thoughtful review.

**Reviewer #3:**

Q1. Maybe figure illustrating all the specific methods/functions/packages and how they relate to each other would be helpful.

Response: Thank you very much for valuable comments, which will improve the quality of our manuscript greatly. We have added the corresponding parts in Figure 1 to illustrate the methods and relationship in our study.

Q2. As for the methods, numerous packages are mentioned as being used. What would be additionally helpful would be to know exactly which functions without those packages helped produce the analysis. Were the defaults used for each of the functions or where some of the parameters modified.

Response: Thank you for your valuable comments. As for unsupervised clustering, we have added details of the modified parameters for the “ConsensusClusterPlus” package in Lines 168-172. For machine learning, we reintroduce the cross-validation methodology in Lines 200-201, stating that the optimal choice of the four machine learning algorithms is derived from 5-fold cross-validation with default parameters. All analyses were performed with default parameters based on 5-fold cross-validation, and no special customization was made. For the single-cell analysis, we have added the modified parameters of PCA. Please see Lines 249-256. For the cell-cell communication and signaling pathway analysis, we have added that all calculations are performed automatically with default parameters. Please see Lines 267-271. Additionally, we have highlighted the defaults or the modified parameters we have already mentioned in each section of the Methods in yellow.

We hope our revision will address your concern.

Q3. For the ML methods, it would be nice to clarify what the abbreviations stand for before using them (XGB, SVM, GLM, and RF). It is also unclear if any training/testing datasets were used. Information is lacking as to model performance and how that was evaluated.

Response: Thank you for your valuable comments. We apologize for the ambiguity of the article due to our lack of consideration. We used to mention the full name of the four machine learning methods in Lines 103-105. We have now added the full name of the abbreviation in the ML methods section to make it more understandable. Please see Lines 187-189. As for the lack of information on the training/testing datasets, we sincerely apologize for our oversights. We have added the content explaining how the training/testing dataset was utilized to define hub-DECAGs for AD risk. Please see Lines 382-387. For the detailed information and the evaluation of our risk model, we have added more details in our revised manuscript, including the details of the external validation method on Lines 211-214 and confidence intervals of the two external validation datasets in Lines 397-402.

We have revised the manuscript to explicitly detail the ML methods. Thank you once again for your thoughtful review.

Q4. For construction and validation of a risk model, it is unclear what the outcome is and what were the dependent variables. How many samples were used here? Overall, this info (sample size) is lacking for much of the analyses mentioned.

Response: Thank you for your valuable feedback. We apologize for our omission. We have added more details about the AD risk model, including the calculation of the outcome in the nomogram model and the AD risk-predicting variables. Please review Lines 388-394.

We used to mention the details of the datasets in Table S1. Please see Lines 115-122. The risk model construction dataset (GSE63060) includes 145 AD and 104 control blood samples. In machine learning, we randomly assigned AD samples, 30% for the test cohort and 70% for the training cohort. We used two risk model validation datasets: GSE33000, containing 310 AD and 157 control cortex samples, and GSE122063, containing 56 AD and 44 control cortex samples.

We hope these revisions adequately address your concerns.

**Reviewer #4:**

Q1. The title says, "integrated analysis," but the study only uses one dataset (GSE63060) for training. I suggest changing the title unless multiple datasets or data types are combined for analysis.
Response: Thank you very much for raising this point. Although the training set was based on GSE63060, our study indeed incorporates an integrated analysis approach by combining several datasets and diverse data types. In addition to using GSE63060 for model development, we validated our risk model with two external cortical datasets (GSE33000 and GSE122063) and further investigated cell-specific gene expression using a scRNA-seq dataset (GSE181279). Moreover, our analysis spans from bioinformatic integration and machine learning to experimental validation (qPCR), which together provide a comprehensive insight into the role of cuproptosis in Alzheimer’s disease

Q2. The list of DECAGs like COX17, ATP7A, MTF1, etc., is mentioned, but their role in Alzheimer's Disease (AD) isn’t explained well. It would help to discuss how these genes are known to be involved in AD.

Response: Thank you for your constructive suggestion. We have now added the content discussing the roles of the DECAGs in AD. Please see Lines 523-536. Note that in combination with our findings, we have previously discussed the role of the 5 hub-DECAGs in AD. Please see Lines 555-580. We appreciate your attention to detail.

Q3. The enrichment analyses (GO, KEGG) show an overlap with well-known metabolic pathways. It would be useful to explain whether these pathways are already well-known in AD or if the study provides new insights.

Response: Thank you for your valuable suggestion. We have now discussed the relationship of these enrichment pathways and AD pathogenesis to enrich our content. Please see Lines 543-544. We appreciate your thoughtful review.

Q4. The correlation between immune cell infiltration and DECAG expression is interesting, but the manuscript doesn’t fully discuss the impact of immune cell differences between AD patients and healthy controls. It would be useful to explore how these immune changes might affect AD progression.

Response: Thank you for your valuable feedback. In the discussion sections, we added content about the significance of the immune changes in AD patients and the close link between these changes and the DECAGs. Please see Lines 581-596. Thank you for helping us organize our work.

Q5. The molecular docking results are intriguing, but the limitations and assumptions behind the method aren’t discussed. It would be good to mention how the docking results might translate to actual drug effectiveness and what experiments would be needed to confirm these findings.

Response: Thank you for your valuable comments regarding the molecular docking results. We appreciate your insights and have revised the discussion section on molecular docking to make it more comprehensive. Please see Lines 648-657. Thank you once again for your thoughtful review.

Q6. The study uses several machine learning algorithms (XGB, GLM, RF, SVM) to predict outcomes, but there is no clear explanation on how the models were tuned or selected. Also, it’s unclear whether any validation methods like cross-validation were used to check the model's reliability.

Response: Thank you for your valuable feedback. We apologize for the ambiguity of the article. We have rewritten the results section “Construction and validation of a risk model” and added much content, including explaining how the training/testing dataset was utilized to define hub-DECAGs for AD risk and the calculation of the outcome in the nomogram model and the AD risk-predicting variables. Please see Lines 388-394. We validated the risk model using ROC curves on external datasets. We have added the validation method in Lines 211-214 and revised the validation results in Lines 397-402.

Thank you once again for your thoughtful review.

Q7. The manuscript mentions using "Seurat" for scRNA-seq analysis, but it doesn’t explain how data quality was checked or how noise was reduced beyond general normalization and batch correction. More details would be helpful.

Response: Thank you for your constructive suggestion. We apologize for our omission. We have now added more details about quality checking and noise reduction, as shown below:

Firstly, we add the scatter plot of the correlations of sequencing depth in Figure S2B. A high positive correlation (0.92) indicates that cells with higher sequencing depth (more UMIs) tend to detect more genes. A near-zero correlation (-0.01) suggests that sequencing depth is independent of mitochondrial gene expression. All of these indicate the good quality of our scRNA data. Please see Lines 449-452

Secondly, we expanded the single-cell analysis methods section to make the PCA and clustering analyses more detailed. Please see Lines 249-256.

Thirdly, we added four figures about PCA in Figure S2. An elbow plot of the principal components in PCA suggests that selecting the first 20 components for further analysis is appropriate (Figure S2C). A two-dimensional PCA plot showing the projection of the data onto the first two principal components (PC1 and PC2), revealing the similarities and grouping structures among samples (Figure S2D). The feature genes of each principal component are displayed in Figures S2E and F, preparing for accurate clustering analysis. Please see Lines 453-455.

We hope these revisions adequately address your concerns

Q8. The manuscript states that the five hub genes were identified using machine learning (ML), but this seems to be incorrect. The hub genes were actually identified by analyzing the protein-protein interaction (PPI) network, specifically using degree analysis to find genes with the most interactions. I suggest changing Figure 1 and the methods section to make it clear that the hub genes were found using the PPI network and degree analysis, not ML.

Response: Thank you very much for your constructive comments. In our study, the five hub genes were selected through a machine learning pipeline that incorporated four different algorithms (XGB, GLM, RF, and SVM) to identify the genes most predictive of AD risk. The machine learning approach allowed us to rank the differentially expressed cuproptosis-associated genes based on their performance metrics, with the top five candidates (FDX1, GLS, MAP2K1, PDK1, and SOD1) chosen for further analysis.

It is important to clarify that while we constructed a PPI network to visualize the interactions among all the differentially expressed genes and to provide insight into potential functional relationships, this network was not the basis for the identification of the hub genes. We have revised both Figure 1 and the Methods section to explicitly distinguish between the machine learning-based hub gene selection and the complementary PPI network analysis. Please refer to the revised Figure 1 and Lines 140-143 in the revised manuscript.

We sincerely appreciate your suggestions, which have helped improve the clarity of our manuscript.
