# Supplementary material for: Novel insights from comprehensive analysis: The role of cuproptosis and peripheral immune infiltration in Alzheimer’s disease
Source: PLoS One. 2025 Jun 25;20(6):e0325799. doi: 10.1371/journal.pone.0325799 (PMC12194219; doi:10.1371/journal.pone.0325799)
Supplement: Attachment — Submitted filename: Response_to_Reviewers_auresp_2.docx [file pone.0325799.s015.docx]

Dear Editor and Reviewer,

We appreciate your permission to revise the manuscript again. Thank you very much for your kind, positive, and constructive comments on the manuscript. All journal requests were taken seriously. After carefully reading your comments and suggestions, we have revised and responded according to them and displayed them below:

**Reviewer #4:**

Q1. The response provided by the authors to the concern about the title and "integrated analysis" approach is not accurate. The title, "Novel insights from integrated analysis," implies that multiple datasets were integrated during model development or training, which is not the case here. The study only uses GSE63060 for model training, and the additional datasets are used only for validation and subsequent analysis.

To maintain scientific accuracy, I strongly suggest revising the title to reflect the actual approach used in this study. The term "integrated analysis" should refer to the integration of multiple datasets during the model construction phase, not just the validation or post hoc analyses.

Response: We sincerely appreciate your patience in explaining all this to us. We have now revised “integrated” to “comprehensive” to make our title more accurate. Please see Lines 4. We also acknowledged the potential limitations of single-dataset training in the revised discussion. Please see Lines 691-693: although our study provided a strong relationship between CAGs and AD in the peripheral immune system using multi-omics studies, the bulk and single-cell RNA data were obtained from a limited number of samples.

Q2. While the authors have addressed the similarities with previous studies, the significant overlap between this manuscript and the previous studies warrants a more detailed comparison. It would be beneficial for the authors to directly compare their results with the findings from these studies:

1. <https://doi.org/10.3389/fnagi.2022.932676>

2. <https://doi.org/10.3389/fnagi.2023.1204530>

By doing so, the authors can more clearly identify any common findings or discrepancies, strengthening their claim of novelty and providing further evidence of how their approach differs or adds to existing knowledge in the field. This would also help clarify whether their conclusions, such as those regarding cuproptosis in AD progression, are consistent with or diverge from previous studies.

Response: We sincerely thank you for highlighting the importance of contextualizing our findings within existing literature. We would like to address these points as follows:

1.Direct Comparison with Lai et al. (2022, DOI: 10.3389/fnagi.2022.932676)

Both studies confirmed the dysregulation of copper homeostasis in patients with Alzheimer's disease (AD). Unlike their cortex tissues data, we also observed elevated copper levels in peripheral blood.

While Lai et al. primarily linked copper imbalance to amyloid-β aggregation, our work explicitly establishes cuproptosis as a driver of neuronal loss via key DECAGs- -mediated dysfunction. Moreover, the content in these pictures is completely different from other studies, including the enrichment analysis, GSVA analysis in the two Clusters, machine-learning models, single-cell analysis, cellular communication, and Pet-CT results.

2. Direct Comparison with Nie et al. (2023, DOI: 10.3389/fnagi.2023.1204530)

Both studies identify cuproptosis genes as key regulators in AD progression. However, our study differs in several important respects: **a.** Unlike their single-cohort analysis, we employed cross-dataset validation (GSE63060 training → GSE33000/GSE122063/GSE181279 external testing) **b.** Unlike previous studies, we have incorporated single-cell analysis to a stronger, more targeted hypothesis regarding cuproptosis involved in AD progression. **c.** The number of CAGs included in this study was greater than other studies, and we employed four machine-learning methods to develop a cuproptosis-related model, which was distinguished from other work. **d.** Our interpretation of the results diverged from that in the referenced studies. We provided a detailed discussion on how our findings contribute to a deeper understanding of MIF pathway and intracellular communication and the potential implications for future research.

We have revised the **Discussion** section to ensure clarity regarding our novel findings, which also underscore our study's unique contributions while contextualizing them within existing literature. Please see Lines 673-690. We appreciate the opportunity to clarify our work’s unique position in the field and welcome further suggestions.
