# Supplementary material for: Novel insights from comprehensive analysis: The role of cuproptosis and peripheral immune infiltration in Alzheimer’s disease
Source: PLoS One. 2025 Jun 25;20(6):e0325799. doi: 10.1371/journal.pone.0325799 (PMC12194219; doi:10.1371/journal.pone.0325799)
Supplement: Attachment — Submitted filename: Response_to_Reviewers_auresp_3.docx [file pone.0325799.s016.docx]

Dear Editor and Reviewer,

We sincerely appreciate your permission to revise the manuscript once again. Thank you very much for your kind, positive, and constructive feedback, which has been invaluable in improving our work. We have carefully reviewed all the journal’s requests and have given them our utmost attention. After thoroughly considering your comments and suggestions, we have meticulously revised the manuscript accordingly. Below, we have provided a detailed response to each point, outlining the changes made to address your recommendations.

**Response to Academic Editor:**

Please review your reference list to ensure that it is complete and correct. If you have cited papers that have been retracted, please include the rationale for doing so in the manuscript text, or remove these references and replace them with relevant current references. Any changes to the reference list should be mentioned in the rebuttal letter that accompanies your revised manuscript. If you need to cite a retracted article, indicate the article’s retracted status in the References list and also include a citation and full reference for the retraction notice.

Response: Thank you for your kind and constructive reminder of our work on referencing. Based on your comments, we have carefully reviewed the reference list and confirmed that all articles in the References have met the criteria for ‘cannot be retracted’ status. We sincerely appreciate your valuable comments.

**Response to Reviewer #4:**

Q1. Thank you to the authors for addressing the comments. However, I would kindly suggest improving the quality of Figures 8, 9, and 11, as they currently appear unclear. Enhancing their quality would significantly improve clarity and readability.

Response: Thank you for your insightful comments on the quality of Figures 8, 9, and 11, which will improve the integrity of our study greatly. We have Enhanced the quality of these figures and displayed them in PDF format to improve clarity and readability of our study based on your advice. We hope these revisions adequately address your concerns. Thank you once again for your thoughtful review.
